# Supplementary material for: The effect of an mHealth application based on continuous support and education on fear of childbirth, self-efficacy, and birth mode in primiparous women: A randomized controlled trial
Source: PLoS One. 2023 Nov 1;18(11):e0293815. doi: 10.1371/journal.pone.0293815 (PMC10619799; doi:10.1371/journal.pone.0293815)
Supplement: S1 File — (DOCX) [file pone.0293815.s001.docx]

**Research protocol**

**Project summary**

Fear of childbirth (FOC) is a risk factor for maternal and child adverse outcomes and needs to be addressed during pregnancy. In this study, we aim to determine the effect of an mHealth application based on continuous support and education on FOC, self-efficacy, and birth mode in primiparous women. This study will be a single-center, Randomized Controlled Trial (RCT). It will conduct on seventy primiparous pregnant women with FOC attending a university-dependent governmental hospital in Tehran, Iran. Women who meet the inclusion criteria will randomly assign to two parallel intervention and control groups. Study participants in the intervention group will receive an mHealth application named Tele-midwifery, an interactive application based on multipurpose approaches, such as education and consultation, emphasizing continuous care for eight weeks. Women in the control group will receive routine prenatal care. FOC and self-efficacy will be measured via Wijma Delivery Expectancy/Experience (W-DEQ) questionnaire and Childbirth Self-efficacy Inventory (CBSEI), respectively. Data will be collected at the beginning of the study, 36 weeks of pregnancy, and after childbirth. Data will be analyzed by SPSS-25 using Chi-square, Fisher’s exact test, independent t-tests, and repeated-measures analysis of variance. We hypnotize that the intervention in this study will improve the FOC score, increase self-efficacy, and lower the number of C-sections.

**General information**

**Protocol title:**

The Effect of an mHealth Application based on Continuous Support and Education on Fear of Childbirth, Self-efficacy, and Birth Mode in Primiparous Women: A Randomized Controlled Trial

**Authors:**

Sahar Khademi, MSc, School of Nursing and Midwifery, Tehran University of Medical Sciences, Sahar.khademi975@gmail.com,

Elham Ebrahimi, Ph.D. of Reproductive Health, School of Nursing and Midwifery, Tehran University of Medical, ebrahimi_308@yahoo.com,

Ahmad Khosravi, Ph.D. of Epidemiology, Center for Health-Related Social & Behavioral Sciences, Shahroud University of Medical Sciences, khosravi2000us@yahoo.com,

Shohreh Movahedi Fellowship in infertility, Department of Obstetrics & Gynecology of Baharlu hospital, Tehran University of Medical Sciences, shmovahedy@razi.tums.ac.ir,

**Name and address of the hospital involved in the research:**

Baharloo hospital, Tehran, Iran

**Rationale and background information**

Fear of Childbirth (FOC) affects almost 14 percent of women severely and there is an increasing trend in the number of women experiencing FOC during their life course (1). Women suffering from FOC usually experience nightmares, physical complaints, and a lack of focus on work or social life (2). FOC is related to increased emergency and elective C-sections (CS) (3). Moreover, women with FOC experience more complications such as preterm birth, prolonged labor, dystocia, postpartum depression, post-traumatic stress disorder, and less mother-child bonding (4-8). FOC is strongly associated with a lack of childbirth self-efficacy, especially in nulliparous women; therefore, the effort to increase self-efficacy is a practical part of a successful strategy to improve FOC (9). Some interventions have been shown to be effective; however, there is no consensus regarding the best treatment for alleviating women's fears. Additionally, most of these interventions are in-person and are related to high costs and difficulty in implementation (7). Due to these challenges, digital methods such as mHealth applications have gained attention in various fields to improve women's health. Receiving pregnancy-related services including information and consultation by digital technologies such as mHealth applications are shown to be acceptable by pregnant women, leading to positive pregnancy and childbirth experiences (10). Receiving health care through mHealth, make patients more responsible for obtaining information and learning; thus, more in-depth knowledge and awareness would acquire, which are factors related to reducing FOC and increasing self-efficacy (11). The application of mHealth interventions to improve the FOC and its related factors including self-efficacy and birth mode have received less attention among Iranian pregnant women. This method may reduce the FOC, increase women's self-efficacy and the number of CS. Therefore, in this study, an mHealth application will consider a possible strategy for treating women with FOC.

**Study goals and objectives**

1) Women receiving the Tele-midwifery intervention will report lower levels of FOC compared to the control group.

2) Women receiving the Tele-midwifery intervention will have a higher self-efficacy score compared to the control group.

3) Women receiving the Tele-midwifery intervention will have a lower number of CS compared to the control group.

**Study design**

This study will be is an RCT with two groups of control and intervention, and will be conduct on seventy primiparous women who will attend the prenatal clinic of one of the public university hospitals in Tehran, Iran (Baharlou), to receive prenatal care from February to April 2020.

**Inclusion criteria**:

Inclusion criteria in this study will be: being primiparous, having FOC (confirmed by the score of 38 and above on the Wijma Delivery Expectancy/Experience Questionnaire (W-DEQ)), being aged between 18 to 40 years, being pregnant with a singleton fetus, being in 26-29 weeks of pregnancy, having access to a smart device such as smartphone, or a tablet (women or their spouses), as well as the ability to work with them, having access to the internet, not having chronic diseases and CS indications before or during pregnancy.

**Exclusion criteria:**

The exclusion criteria will be, emergency pregnancy conditions that require further medical interventions (e.g., placental abruption, fetus abnormalities, preeclampsia).

### Methodology

**Outcomes**

The primary outcome of this study will be change in FOC score, and the secondary outcomes will be change in childbirth self-efficacy score, and birth mode.

**Measures**

The FOC will be measured using W-DEQ versions A and B. W-DEQ version A evaluates women's prenatal expectances before childbirth, and W-DEQ version B evaluates experiences with recent childbirth. Each version contains 33 items with a 6-point Likert scale ranging from 0 (extremely) to 5 (not at all), and scores range from 0 to 165, with higher scores indicating higher fear of childbirth (12). According to this questionnaire, a score less than or equal to 37 represent mild fear, a score of 38-65 represents a moderate level, 66-84 represents high level of fear, and a score more than 85 shows a severe level of fear. The reliability of the W-DEQ version A and B questionnaire for nulliparous women was confirmed with Cronbach's alpha of 0.89 and 0.92, respectively (12). The validity and reliability of the Persian version of this questionnaire for Iranian women have been confirmed (13).

The Childbirth Self-Efficacy Inventory (CBSEI) is a self-report instrument that measures outcome expectancies (OE) and efficacy expectancies (EE) for coping with an approaching childbirth experience. This questionnaire has two parts and 62 items that scored on a ten-point Likert scale (1= not at all; 10= completely sure), with a higher score indicating greater childbirth self-efficacy. The reliability of the questionnaire has been confirmed with Cronbach's alpha 0.86-0.95 (14). The validity and reliability of the Persian version of this questionnaire were assessed by Khorsandi et al. (2008). In their study, high internal consistency with Cronbach's alpha coefficient of 0.84 to 0.91 was reported for the CBSEI (15).

**Procedures**

A convenient sampling approach will be used, and a consecutive sample of pregnant women attending the selected prenatal clinic (located in Baharlou hospital) for receiving their routine prenatal care will be evaluated by their medical records for inclusion and exclusion criteria, by a colleague not involving in the study. Afterward, the objectives and goal of the study will be explained to eligible pregnant women, and after filing the consent form, they will be asked to complete the W-DEQ version A. women with a score of 38 and above (moderate and high FOC score) will be enrolled the study.

A randomized allocation into two groups of intervention and control with a 1: 1 ratio will be taken place during the routine prenatal visit in the hospital by a colleague who will be not involved in the study and computerized generated random sequences. In both intervention and control groups, the Tele-midwifery application will be installed on their smartphones during their prenatal visit. This application will only allow access to the questionnaires in the post-test, and follow-up stages for the participants in the control group. To prevent information contamination to the control group, after installing the application and before activating their account, women must select their group type as A or B. In addition, we will ask women in both groups to avoid sharing the contents of the application with each other for the duration of the trial. The Tele-midwifery application have two main stages for answering the questions. After filling the baseline questionnaire in the initial registration, including demographic, W-DEQ, and CBSE questionnaires, each group’s participants will complete the W-DEQ and CBSE questionnaires again at 34-36 weeks (after eight weeks of receiving the intervention). Participants will be also asked to inform the researcher by sending a message after hospitalization for their birth, whether in the Tele-midwifery application or by contacting the researcher directly by her phone number. Afterward, the W-DEQ version B questionnaire will appear in the application within the first two hours after birth, and the birth mode will be recorded. The investigators, care providers, data collectors, and statisticians will be masked from the allocation.

**Dimensions of intervention:**

**Education**

The educational content of the Tele-midwifery application will be designed to include all possible causes of FOC in Iranian women, such as fear of labor pain, lack of information related to the mother and baby's health, what women should expect during labor and birth, the characteristics of each stage of labor, misconceptions such as distrust of healthcare providers, and complications of vaginal birth (16, 17). The educational content will be included information and exercises to increase women’s knowledge and challenge their underlying beliefs causing the FOC. Besides the educational information will be in form of text, audio, video, and image.

**Continuous support and engagement of women:**

### To provide continuity of care between two prenatal visits and support participants in the intervention group, they can contact the researchers if they have concerns and questions about their pregnancy and birth care. The team of researchers including midwives and obstetricians will be available throughout the day (between 7 am to 9 pm), to provide women with an accessible source of information about their pregnancy and childbirth decisions. They can also write about their feelings and experiences publicly in the forum linked to the educational content, and pregnant women will have the opportunity to exchange their feelings with each other under the research team's supervision.

### The control group will receive routine prenatal care. Currently, there is no specific guidelines in Iran, for providing care to pregnant women suffering from FOC. Routine prenatal care includes regular visits to monitor women and fetal health. To better access women in the control group, we will ask them not to uninstall the application from their mobile phones until birth and answer questionnaires after eight weeks and after childbirth.

### Data management and statistical analysis

The data will be analyzed using SPSS software version 25, and p-value > 0.05 will consider a statistically significant level. First, the Kolmogorov‐Smirnov test will use to examine the normal distribution of data. After determining the normal distribution of data, to compare statistical differences of demographic data in two groups, an independent t-test, Chi-square test, and Fisher's exact test will be used. Also, for analyzing outcome variables, **r**epeated-measures analysis of variance (ANOVA) with a pairwise comparison of groups with Bonferroni correction, independent t-test, and Chi-square will determine the differences between the groups. **Sample size**

The sample size was calculated based on the results of Gözde Birsbir et al. (2016) study (18). A sample size of 70 pregnant women (35 in each group) was estimated. A two-sided t-test of difference between means with the power of 90%, the type one error equal to 0.05, mean difference of 21 between groups, and standard deviation of 7.2 was consider calculating the sample size. We accounted 20% attrition rate in estimating the sample size.

**Expected outcomes of the study**

The proposed project evaluates an innovative mHealth intervention that uses evidence-based content with a comprehensive understanding of pregnancy and childbirth. Our intervention is based on technology that has advantages such as high accessibility, and flexibility in terms of time and location for pregnant women which can be a convenient way of providing care. The findings of this study will contribute to an improvement in maternal mental health which can be used for supportive treatment for women diagnosed with FOC.

**Duration of the project**

We predict six months to conduct the study. The first three months will be required for recruitment and intervention and for collecting data. Then in the next stage, with the final data collected, we will complete data analysis, and reporting, and prepare a manuscript.

**Project management**

SM, SK, and EB will participate in developing the tele-midwifery intervention. AK and SK will analyze the data, and AK, and SK will help interpret the results. EE, and SK will write the manuscript and EE, AK and SM will review it for critical intellectual content.

**Ethics**

The research protocol has been reviewed and received Human Research Ethics Committee Approval from Tehran university of medical sciences (IR.TUMS.FNM.REC.1398.135).

**Trial registration:**

IRCT registration number: IRCT20200122046227N1, Registered on 27 January 2020.

**References**

1. O'Connell MA, Leahy‐Warren P, Khashan AS, Kenny LC, O'Neill SM. Worldwide prevalence of tocophobia in pregnant women: systematic review and meta‐analysis. Acta obstetricia et gynecologica Scandinavica. 2017;96(8):907-20.

2. Wijma K, Wijma B, editors. A Woman Afraid to Deliver: How to Manage Childbirth Anxiety2017.

3. Stoll K, Edmonds JK, Hall WA. Fear of Childbirth and Preference for Cesarean Delivery Among Young American Women Before Childbirth: A Survey Study. Birth. 2015;42(3):270-6.

4. Adams SS, Eberhard-Gran M, Eskild A. Fear of childbirth and duration of labour: a study of 2206 women with intended vaginal delivery. BJOG. 2012;119(10):1238-46.

5. Dencker A, Nilsson C, Begley C, Jangsten E, Mollberg M, Patel H, et al. Causes and outcomes in studies of fear of childbirth: A systematic review. Women and birth : journal of the Australian College of Midwives. 2019;32(2):99-111.

6. Laursen M, Johansen C, Hedegaard M. Fear of childbirth and risk for birth complications in nulliparous women in the Danish National Birth Cohort. BJOG: An International Journal of Obstetrics & Gynaecology. 2009;116(10):1350-5.

7. Serçekuş P, Okumuş H. Fears associated with childbirth among nulliparous women in Turkey. Midwifery. 2009;25(2):155-62.

8. Areskog B, Uddenberg N, Kjessler B. Postnatal emotional balance in women with and without antenatal fear of childbirth. J Psychosom Res. 1984;28(3):213-20.

9. Salomonsson B, Gullberg MT, Alehagen S, Wijma K. Self-efficacy beliefs and fear of childbirth in nulliparous women. J Psychosom Obstet Gynaecol. 2013;34(3):116-21.

10. Sandall J, Soltani H, Gates S, Shennan A, Devane D. Midwife-led continuity models versus other models of care for childbearing women. The Cochrane database of systematic reviews. 2016;4:Cd004667.

11. Çankaya S, Şimşek B. Effects of Antenatal Education on Fear of Birth, Depression, Anxiety, Childbirth Self-Efficacy, and Mode of Delivery in Primiparous Pregnant Women: A Prospective Randomized Controlled Study. Clin Nurs Res. 2020:1054773820916984.

12. Wijma K, Wijma B, Zar M. Psychometric aspects of the W-DEQ; a new questionnaire for the measurement of fear of childbirth. J Psychosom Obstet Gynaecol. 1998;19(2):84-97.

13. Mortazavi F. Validity and reliability of the Farsi version of Wijma delivery expectancy questionnaire: an exploratory and confirmatory factor analysis. Electron Physician. 2017;9(6):4606-15.

14. Lowe NK. Self-efficacy for labor and childbirth fears in nulliparous pregnant women. Journal of Psychosomatic Obstetrics & Gynecology. 2000;21(4):219-24.

15. Khorsandi M, Ghofranipour F, Faghihzadeh S, Hidarnia A, Bagheban AA, Aguilar-Vafaie ME. Iranian version of childbirth self-efficacy inventory. Journal of clinical nursing. 2008;17(21):2846-55.

16. Kananikandeh S, Amin Shokravi F, Mirghafourvand M, Jahanfar S. Factors of the childbirth fear among nulliparous women in Iran. BMC Pregnancy and Childbirth. 2022;22(1):547.

17. Andaroon N KM, Kimiaei SA, Esmaeili H. . Relationship between Intensity of fear of Childbirth with choosing mode of delivery in Primiparous Women. Iran J Obstet Gynecol Infertil 2017;20(5):68-75.

18. Gökçe İsbir G, İnci F, Önal H, Yıldız PD. The effects of antenatal education on fear of childbirth, maternal self-efficacy and post-traumatic stress disorder (PTSD) symptoms following childbirth: an experimental study. Applied Nursing Research. 2016;32:227-32.
