# Supplementary material for: The effect of an mHealth application based on continuous support and education on fear of childbirth, self-efficacy, and birth mode in primiparous women: A randomized controlled trial
Source: PLoS One. 2023 Nov 1;18(11):e0293815. doi: 10.1371/journal.pone.0293815 (PMC10619799; doi:10.1371/journal.pone.0293815)
Supplement: S2 File — (DOC) [file pone.0293815.s002.doc]

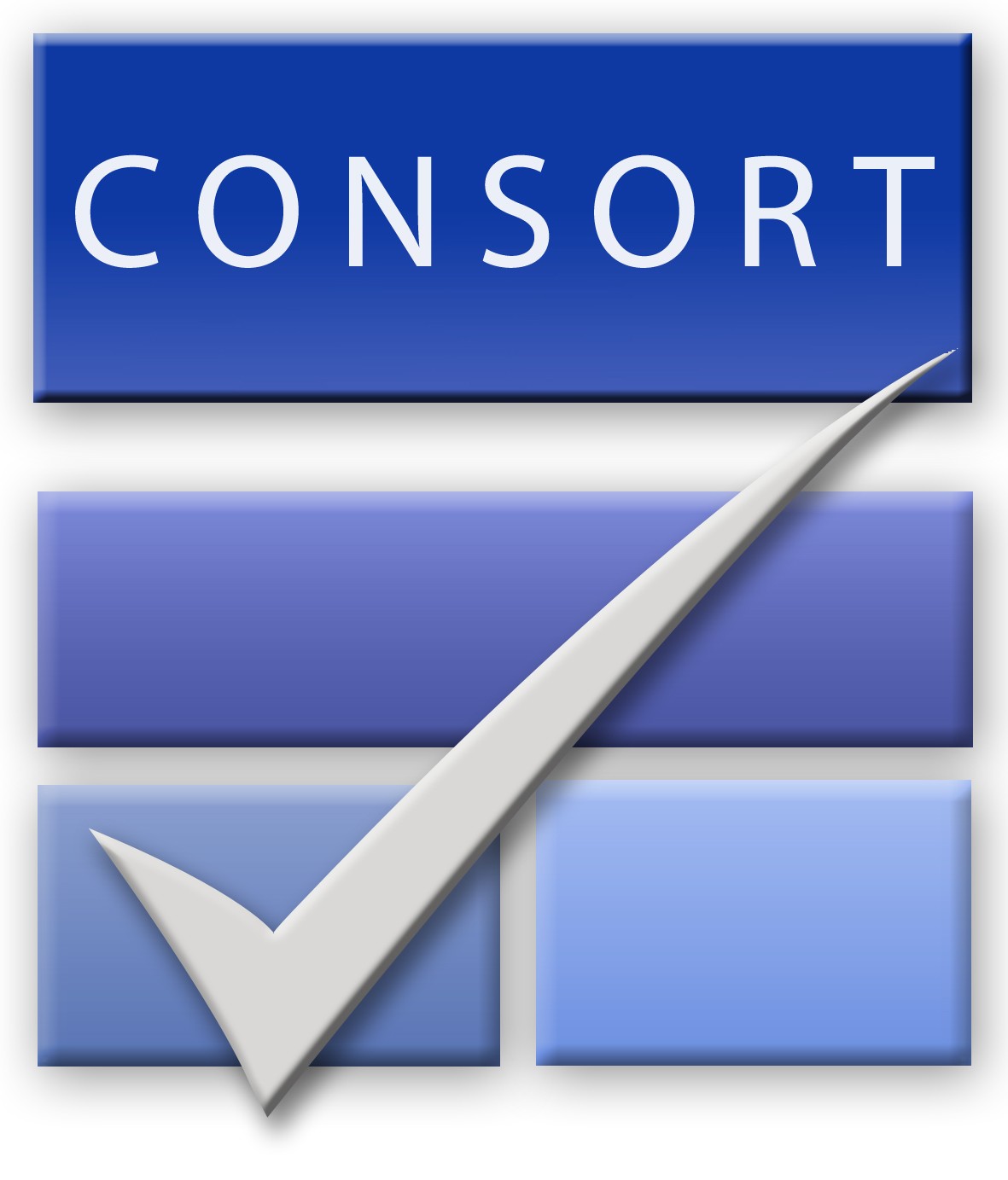
 CONSORT 2010 checklist of information to include when reporting a randomised trial*

| Section/Topic | Item No | Checklist item | Reported on page No | |
| --- | --- | --- | --- | --- |
| Title and abstract | | | | |
|  | 1a | Identification as a randomised trial in the title | | 1 (Title) |
|  | 1b | Structured summary of trial design, methods, results, and conclusions (for specific guidance see CONSORT for abstracts) | | 2 (Abstract) |
| Introduction | | | | |
| Background and objectives | 2a | Scientific background and explanation of rationale | | 3,4 (Introduction) |
|  | 2b | Specific objectives or hypotheses | | 4,5 (Last paragraph in the introduction and first paragraph in the method section) |
| Methods | | | | |
| Trial design | 3a | Description of trial design (such as parallel, factorial) including allocation ratio | | 7,8 (Starting from second paragraph in Procedures subheading) |
|  | 3b | Important changes to methods after trial commencement (such as eligibility criteria), with reasons | | N/A |
| Participants | 4a | Eligibility criteria for participants | | 5 (Second paragraph in Method section) |
|  | 4b | Settings and locations where the data were collected | | 5 (First paragraph in Method section) |
| Interventions | 5 | The interventions for each group with sufficient details to allow replication, including how and when they were actually administered | | 7-10 (Procedures, Tele_midwifery application, and Dimensions of intervention subheadings) |
| Outcomes | 6a | Completely defined pre-specified primary and secondary outcome measures, including how and when they were assessed | | 5,6 (Outcomes and measures subheading) |
|  | 6b | Any changes to trial outcomes after the trial commenced, with reasons | | N/A |
| Sample size | 7a | How sample size was determined | | 6,7 (Sample size subheading) |
|  | 7b | When applicable, explanation of any interim analyses and stopping guidelines | | N/A |
| Randomisation: |  |  | |  |
| Sequence generation | 8a | Method used to generate the random allocation sequence | | 7 (Second paragraph of Procedure subheading) |
|  | 8b | Type of randomisation; details of any restriction (such as blocking and block size) | | 7 (Second paragraph of Procedure subheading) |
| Allocation concealment mechanism | 9 | Mechanism used to implement the random allocation sequence (such as sequentially numbered containers), describing any steps taken to conceal the sequence until interventions were assigned | | 7,8 (From the second paragraph in Procedures subheading to the end of section) |
| Implementation | 10 | Who generated the random allocation sequence, who enrolled participants, and who assigned participants to interventions | | 7 (Second paragraph of Procedure subheading) |
| Blinding | 11a | If done, who was blinded after assignment to interventions (for example, participants, care providers, those assessing outcomes) and how | | 8 (At the end of Procedure subheading) |
|  | 11b | If relevant, description of the similarity of interventions | | N/A |
| Statistical methods | 12a | Statistical methods used to compare groups for primary and secondary outcomes | | 10,11 (Statistical Analyzes) |
|  | 12b | Methods for additional analyses, such as subgroup analyses and adjusted analyses | | N/A |
| Results | | | | |
| Participant flow (a diagram is strongly recommended) | 13a | For each group, the numbers of participants who were randomly assigned, received intended treatment, and were analysed for the primary outcome | | 11 (First paragraph of result section and Figure 1) |
|  | 13b | For each group, losses and exclusions after randomisation, together with reasons | | 7 (First paragraph of Procedures section and Figure 1) |
| Recruitment | 14a | Dates defining the periods of recruitment and follow-up | | 5 (First paragraph of Methods section) |
|  | 14b | Why the trial ended or was stopped | | N/A |
| Baseline data | 15 | A table showing baseline demographic and clinical characteristics for each group | | 20 (Table 2) |
| Numbers analysed | 16 | For each group, number of participants (denominator) included in each analysis and whether the analysis was by original assigned groups | | 11 (First paragraph of result section and Figure 1) |
| Outcomes and estimation | 17a | For each primary and secondary outcome, results for each group, and the estimated effect size and its precision (such as 95% confidence interval) | | 21 (Table 3 and Table 4) |
|  | 17b | For binary outcomes, presentation of both absolute and relative effect sizes is recommended | | 21,11,12 (Result section and Table 3,4) |
| Ancillary analyses | 18 | Results of any other analyses performed, including subgroup analyses and adjusted analyses, distinguishing pre-specified from exploratory | | N/A |
| Harms | 19 | All important harms or unintended effects in each group (for specific guidance see CONSORT for harms) | | N/A |
| Discussion | | | | |
| Limitations | 20 | Trial limitations, addressing sources of potential bias, imprecision, and, if relevant, multiplicity of analyses | | 15 (Strengths and limitations of research) |
| Generalisability | 21 | Generalisability (external validity, applicability) of the trial findings | | 13-15 (Discussion) |
| Interpretation | 22 | Interpretation consistent with results, balancing benefits and harms, and considering other relevant evidence | | 13-15 (Discussion)) |
| Other information | | | |  |
| Registration | 23 | Registration number and name of trial registry | | 2 (At the end of the Abstract) |
| Protocol | 24 | Where the full trial protocol can be accessed, if available | | 2 2 (At the end of the Abstract) |
| Funding | 25 | Sources of funding and other support (such as supply of drugs), role of funders | | 17 (Funding statement) |

*We strongly recommend reading this statement in conjunction with the CONSORT 2010 Explanation and Elaboration for important clarifications on all the items. If relevant, we also recommend reading CONSORT extensions for cluster randomised trials, non-inferiority and equivalence trials, non-pharmacological treatments, herbal interventions, and pragmatic trials. Additional extensions are forthcoming: for those and for up to date references relevant to this checklist, see [www.consort-statement.org](http://www.consort-statement.org/).
