# Supplementary material for: The effect of an mHealth application based on continuous support and education on fear of childbirth, self-efficacy, and birth mode in primiparous women: A randomized controlled trial
Source: PLoS One. 2023 Nov 1;18(11):e0293815. doi: 10.1371/journal.pone.0293815 (PMC10619799; doi:10.1371/journal.pone.0293815)
Supplement: S3 File — (DOCX) [file pone.0293815.s003.docx]

| **Table S1. Effect of mHealth application on FOC score in intervention group compared to control group at different time points adjusted for Age, Education, Income and Baseline Fear (n = 70)** | | | | | |
| --- | --- | --- | --- | --- | --- |
| **Time/ Group** | **Coefficient** | **SE** | **95% CI** | | **P-Value** |
|  |  |  | **LL** | **UL** |  |
| **Baseline** |  |  |  | |  |
| Intervention vs. Control | 2.89 | 1.77 | -0.66 | 6.42 | 0.11 |
| **After 8 weeks** |  |  |  | |  |
| Intervention vs. Control | - 21.57 | 1.41 | -24.39 | -18.75 | <0.0001 |
| **After childbirth** |  |  |  | |  |
| Intervention vs. Control | -31.34 | 1.39 | -34.12 | -28.56 | <0.0001 |
| SE=Standard Error, CI= Confidence Interval, LL= lower Limit, UL= Upper Limit | | | | | |

| **Table S2. Comparison of mean scores of FOC between intervention and control groups at baseline, after 8 weeks and after childbirth (n = 70)** | | | |
| --- | --- | --- | --- |
| **Variable** | **Intervention Group**  **(n = 35)** | **Control Group**  **(n = 35)** | **P value** |
| **FOC** | **Mean ± SD** | |  |
| Baseline | 109.03 ± 6.67 | 106.20 ± 7.50 | 0.10 |
| After 8 weeks | 87.89 ± 7.36 | 109.03 ± 3.85 | <0.0001 |
| After childbirth | 66.20 ± 6.09 | 97.43 ± 4.70 | <0.0001 |

| **Table S3. Effect of mHealth application on Self-efficacy score in intervention group compared to control group at baseline and 8 weeks after intervention adjusted for age, income, education, and baseline self-efficacy (n = 70)** | | | | | | |
| --- | --- | --- | --- | --- | --- | --- |
| **Time/Group** | **Coefficient** | **SE** | **95% CI** | | | **P-Value** |
|  |  |  | **LL** | **LL** | |  |
| **Baseline** |  |  |  | | |  |
| Intervention vs. Control | 3.24 | 1.97 | -0.35 | | 7.55 | 0.10 |
| **After 8 weeks** |  |  |  | | |  |
| Intervention vs. Control | 83.59 | 2.51 | 78.57 | | 88.62 | <0.0001 |
| SE=Standard Error, CI= Confidence Interval, LL= lower Limit, UL= Upper Limit | | | | | | |

| **Table S4. Comparison of mean difference of self-efficacy score and its subscales in two intervention and control groups (n = 70)** | | | |
| --- | --- | --- | --- |
|  | **Intervention Group**  **(n = 35)** | **Control Group**  **(n = 35)** | **P-Value** |
| **Self-efficacy** | **Mean ± SD** | **Mean ± SD** |  |
| Baseline |  |  |  |
| SE | 244.23 ± 9.69 | 231.29 ±7.43 | 0.07 |
| OE | 119.11 ± 6.03 | 115.57 ± 4.51 | 0.01 |
| EE | 118.14 ± 5.43 | 115.29 ± 3.99 | 0.01 |
| After 8 weeks |  |  |  |
| SE | 329.83 ± 11.37 | 244.89 ± 9.05 | <0.0001 |
| OE | 159.11 ± 6.75 | 121.37 ± 5.66 | <0.0001 |
| EE | 170.71 ± 6.04 | 123.09 ± 5.16 | <0.0001 |
| SE: childbirth Self-Efficacy OE: Outcome Expectancies EE: Efficacy Expectancies | | | |
