# Supplementary material for: The effect of an mHealth application based on continuous support and education on fear of childbirth, self-efficacy, and birth mode in primiparous women: A randomized controlled trial
Source: PLoS One. 2023 Nov 1;18(11):e0293815. doi: 10.1371/journal.pone.0293815 (PMC10619799; doi:10.1371/journal.pone.0293815)
Supplement: Attachment — Submitted filename: Response to Reviewers.docx [file pone.0293815.s004.docx]

**Reviewers' comments:**

Reviewer #1:

"Overall, the authors provide a well-conceived, well-presented report of a randomized clinical trial to assess the impact of a mHealth intervention on Fear of Childbirth and other outcomes with important and significant findings. This is an important addition to the literature on interventions that may improve the quality of antenatal care and impact CS rates. There are however, a number of areas that need further clarification or modification.

Background:
First, it would be valuable to have greater contextual information on the setting in which the study took place. For example, at this particular hospital what is:
1. the overall volume of deliveries and cesarean section rate among all women presenting for care and during the period of the study (What was the impact of COVID?).
2. Who usually cares for women during prenatal care, birth and postnatally- midwives, physicians?
3. What is the usual number of ANC visits in general, and more specifically for the participants in the study what number of ANC visits did the intervention and control groups attend (results) ?"

**Authors' Response:** Thank you for your valuable feedback and the thorough review of our manuscript. We highly appreciate your insightful comments.

We acknowledge the importance of providing more conceptual information on the study setting. However, as the other related information is mentioned in the Methods and Discussion section, we also added points raised in numbers 1-3 in a related section to enhance the conciseness and clarity of the manuscript, and we hope these changes are satisfactory. The related sections now read as follows:

"According to the latest statistics available to the public, in Baharlou Hospital, there are approximately 350 births each month, comprising 38% CS (1). Although compared to the pre-pandemic period, the rate of CS increased during the COVID-19 pandemic (2), the high rate of CS in Iran has always been a challenge in the field of maternal health."

"The routine prenatal care in Iran includes up to 8 prenatal visits, which in the study site would be provided to all pregnant individuals by midwives and obstetricians. Routine prenatal care includes regular visits to monitor women and fetal health."

We also explained more about the number of antenatal care (ANC) visits in the study in the following comments.

"Methods:
Why was the intervention limited to women 18-40?"

**Authors' Response**: Our study's primary focus was on low-risk pregnancies. As a result, we excluded adolescent pregnancies (under 18 years old) and pregnancies in older age groups that are at higher risk of pregnancy complications and need medical interventions. Based on the result of this study, the age range of participants included in the analysis was limited to 18-33 years. This age range is considered low risk for pregnancies, and we attribute this to our study's emphasis on primiparous women, who typically have a lower mean age than multiparous women.

"Line 99 …..the authors write with regard to inclusion criteria….." as well as the ability to work with them, having access to the internet, not having chronic diseases and CS indications before or during pregnancy. The exclusion criteria were, emergency pregnancy conditions that required intervention (e.g., severe symptoms of COVID-19, placental abruption, fetus abnormalities, preeclampsia)." Did any of this occur after enrollment? What happened if these developed during the course of the study?"

**Authors' Response:** We specified these exclusion criteria in our protocol, and participants meeting these conditions were to be excluded from the study. The rationale behind these exclusions was that these conditions involve severe complications requiring emergency care, making them unsuitable for our intervention. Additionally, such conditions may lead to heightened anxiety and fear among individuals.

However, none of these conditions occurred among our participants. One possible reason for this is our eligibility criteria, which excluded high-risk pregnancies. In addition, many of these conditions could have arisen before the 26th week of gestation, when the eligibility criteria were assessed, resulting in the non-recruitment of participants who developed these conditions for the study. We have also documented these details in our CONSORT flow diagram (Figure 1).

"Line 202 The authors state that the researchers were available every day 7 am to 9 pm to answer questions posed in the intervention group. It appears that only 2 of the researchers are clinicians, did they respond to all of the questions? 7 days a week?"

**Authors' Response:** Except for our statistician (AK), the other three authors (SK, EE, and SM) are a certified midwife, a doctor of reproductive health (MSc in Midwifery), and an obstetrician and gynecologist. They are fully capable and eligible to address participants' pregnancy-related questions.

One of the most essential concepts of our intervention is continuous care, which is why the researchers offered uninterrupted support throughout the week, and the researchers answered any questions that might have been raised based on the educational content or general questions regarding their pregnancies, such as the number of in-person visits needed or specific types of food that should be avoided, etc.

"Sample size. The authors fail to describe how the data from the 2016 study by Gozde informed the sample size calculation- a basis for underlying prevalence of FOC?"

**Authors' Response:** Thank you for bringing this to our attention. We appreciate your comment and acknowledge that it is necessary to describe how the cited paper was used to estimate the sample size, outcome, and scale used.

The mean difference in fear of childbirth on the Wijma Delivery Expectancy/Experience Questionnaire (W-DEQ) in the intervention and control group in the cited paper was used to estimate the sample size. We have updated the manuscript and included this information.

"Results:
The authors do not present the number of ANC visits attended in each group. Was there any difference in ANC attendance and may that have been a factor in outcomes? If overall there were very few in person visits in both groups due to COVID, then this study does not actually assess impact of the intervention vs standard care it is intervention vs decreased care."

**Authors' Response:** This is a very interesting point that you brought up. However, capturing the number of ANC visits was beyond the scope of this study. In addition, we want to clarify that our intervention was not intended to substitute in-person ANC visits; it was completely independent of ANC visits and served a different purpose. While we provided continuous care and addressed participants' pregnancy-related questions, we never offered formal ANC procedures such as prescribing medication, requesting screenings, sonograms, or lab tests. As a result, we assume both groups had a similar chance of missing their ANC visits due to any reason and, more specifically, because of the COVID-19 pandemic.

Additionally, it is essential to consider the study timeline. Our research was conducted from February to April. During almost half of the study, COVID-19 had not completely taken over Iran and affected routine care. However, we confirm that antenatal care was continuously offered in the prenatal clinic of the study site, even during the pandemic.

"Process information. Granted, there may be a secondary manuscript in the works that describes the actual dose of the intervention. However, the access to providers via the application to answer questions and respond to concerns is mentioned but not quantified. It would be useful to know how important this feature may be and how labor intensive. It would be valuable to have a sense of how frequently and for what reasons providers were contacted. Were there any referrals made to emergency services through this? Similarly, there is no data provided regarding the frequency with which women read the messages in a timely fashion and accessed the educational modules."

**Authors' Response**: Thank you for your attention to detail. In this current manuscript, we have presented all the data we collected on this intervention.

Considering the multifactorial pathway of fear of childbirth, our intervention included various components to address the diverse factors contributing to fear in pregnant individuals. During the study's design stage, we aimed for a complex intervention, believing that the successful reduction in fear of childbirth resulted from all the intervention components working together. At this stage of testing this new intervention, our primary goal was not to examine the isolated effects of each component, including education and continuous support provided by a care provider to answer questions.

It is important to clarify that this randomized controlled trial was conducted as part of a master's degree thesis with limited budget and time constraints, which unfortunately did not allow us to expand our work and collect more data. However, we totally agree on the importance of collecting more data on each aspect of the intervention and now that the results of this work have shown the possibility of success in reducing fear of childbirth, further comparative research are needed to build upon these findings to determine the most successful component, labor intensiveness (cost-effectiveness) and even explore women's perspectives through qualitative research before implementing the intervention in real-world settings. As previously mentioned, our intervention was not intended as an alternative to antenatal care, but rather as an addition to it. Similar to any educational content our intervention also needed a feature to answer questions raised by our educational material.

Furthermore, although we were prepared to refer participants in case of an emergency, no such contacts were made during the study period. We added this information to the manuscript.

Lastly, in our manuscript, we have addressed your last question:

"The application's educational content was designed for eight weeks in a way that between 3-4 short messages were sent to women on a daily basis." and "The researcher could track the women's activities in the application, and in case the women had not use the application for more than two days, a reminder was sent to follow them up."

"It would be valuable to have an overall sense of birth outcomes among the participants. Live births, stillbirths, early neonatal deaths, preterm birth"

**Authors' Response:** It would indeed be valuable information and an addition to the literature, making it a potential focus for future studies. However, it is important to note that the child outcome was not the objective of this study. Our main emphasis was on maternal outcomes, specifically fear of childbirth, self-efficacy, and childbirth mode. Child outcomes were not listed from the beginning in our ethics approval and protocol, and as a result, were not captured in our data.

"The authors maintained commendable follow up with no losses. I wonder what happened to women who developed pregnancy complications after enrollment- I presume they were kept in the intervention arm? Was this comparable in the groups?"

**Authors' Response**: As mentioned in the first paragraph of the Results section and illustrated in our CONSORT diagram, we did not experience any loss to follow-up or exclusion due to severe pregnancy complications requiring further medical attention and all participants who were initially enrolled were included in the final analysis. Our reasoning behind this decision was explained earlier in response to your previous question regarding exclusion criteria.

"Discussion.

Is there any information on the indications for CS? The authors rightly point out that the underlying CS rate is far above that considered appropriate for primiparous women. How might this have impacted the success of the intervention. Are there other interventions or movement to address the high CS rate in this setting? How might, or did COVID, impact this?"

**Authors' Response**: Thank you for raising this interesting point. The C-section rate in Iran has seen a significant increase over the years, rising from 35% in 2005 to 48% in 2014 and even more in private settings (3), which is considerably high compared to the WHO recommendation. This high rate of C-sections is a critical healthcare issue in the system and has been the subject of investigation and interventions to address this concern. For example, in governmental hospitals, elective C-sections are not possible based on maternal request. However, at the time of our study, no other intervention was ongoing with the purpose of reducing C-sections at the study site. It is important to note that the high rate of C-sections is not specific to our study setting; rather, it is prevalent in many hospitals. Considering this context, we view our intervention as successful in reducing the number of C-sections in the intervention group compared to the control group.

As our study is a governmental hospital, the indication for primary C-sections is fetal distress or maternal emergencies. However, recording the specific indication of C-sections study was beyond the scope of our study.

Regarding the impact of COVID-19 on the C-section rate, it is essential to clarify that none of our participants suffered from COVID-19 infection, and they would have been excluded if they had. Therefore, based on the results of this study, we cannot draw conclusions about the specific impact of COVID-19 on the C-section rate.

Nonetheless, we agree that this could be an interesting and multifactorial aspect to investigate in future research, and we have included it as an implication for future studies in our manuscript.

"I am curious about the impact of the access to providers for questions and concerns. Is it possible to tease out how important the access compared to the educational components is?"

**Authors' Response**: As we comprehensively explained above, we believe our intervention is effective as a composite of different components and did not capture the effect of each component separately. However, we strongly suggest future studies take this into consideration and have added this concern to our implications section in the manuscript.

"The authors to not share their opinions on how this study should inform future work or if there are plans to study this intervention at scale in more of a real world setting. Granted this worked in a highly controlled setting where researchers were available to respond to questions but it is unclear what would happen in a scaled intervention setting."

**Authors' Response**: Based on the findings of our study and your valuable comments regarding other variables that are better to consider when investigating the effect of this intervention, such as access to ANC, child outcome, and each component of the intervention, we added a section under the implications for future research to inform researchers.
We also provided information for discussing the limitations and considerations for scaling up the intervention, including how the availability of researchers in the controlled setting might differ from a real-world scenario.

**"Implications for future research**

In this study, participants received continuous support for addressing their queries throughout the week. However, replicating such personalized attention on a larger scale could be challenging. Real-world implementation might lack sufficient research personnel, potentially affecting intervention quality and adherence. Future research should investigate each component of our study intervention. Additionally, strategies to bridge the gap between controlled settings and real-world scenarios during scaling up need to be explored to maintain intervention quality.

Future studies are also needed to investigate the effects of this study's intervention by considering additional variables such as the number of antenatal care visits and child outcomes, including stillbirth and preterm birth. Recruiting multiparous and primiparous into future research can also be another area before implementing this study intervention in real settings."

"Data:
The authors do not make the data available."

**Authors' Response**: We confirm that all the data collected in this study has been thoroughly presented within the manuscript, including the text, tables, and figures.

However, as the study was conducted in a single center and within a relatively short timeframe, there is a potential risk of participant identification if the data set will make publicly available. Considering the PLOS ONE policy, "For studies involving human research participant data or other sensitive data, we encourage authors to share de-identified or anonymized data. However, when data cannot be publicly shared, we allow authors to make their data sets available upon request", due to the restrictions regarding participant identification, the data set will be available upon request from the corresponding author.

Reviewer #2:

"This manuscript reports the results investigating the effect of an mHealth application based on continuous support and education on primiparous women who were fear of childbirth on self-efficacy and birth mode with a randomized controlled study. I have below minor comments.

Please make a clear statement about which measurement or outcome was used for sample size estimate. How to come with the difference of 21 and sd of 7.2 for that outcome? Pleas note, since the difference of 21 is very big but the variation is small, the n=35/group is far greater than a sample size that achieves 90% power at alpha=0.05."

**Authors' Response**: We appreciate your attention to detail and input regarding the sample size estimation. The cited paper used an intervention similar to our study's intervention; therefore, we used their study as a basis for calculating the minimum fear of childbirth score difference between the intervention and control groups. According to Gözde Birsbir et al.'s (2016), the difference in FOC between groups post-intervention was 21 scores (m i = 25.5, m c= 46.5).

With regard to your second point, we appreciate that you captured this point. The SD mentioned in the manuscript was the differences of the SD between the two groups based on the study used for sample size calculation (s i=18.2 , s c=25.4). We have revised the manuscript to eliminate any confusion and provide a clearer statement regarding the sample size calculation.

Also, our sample size calculation is noted below:

Given the:

α=0.05

β=0.9

μ1=25.5

μ2=46.5

s1=18.2

s2=25.4

We calculate the sample size using the below formula.

$$n=\frac{{( Z_{1-\frac{\alpha}{2}}+ Z_{1-\beta})}^{2}. (S_{1}^{2}+S_{2}^{2})}{\left( \mu_{1}- \mu_{1} \right)^{2}}$$

$$n=\frac{{( 1.96 - (-1.28)}^{2}. ({18.2}^{2}+{25.4}^{2})}{\left( 25.5- 46.5 \right)^{2}}$$

Therefore, we estimated to have 35 participants in each group.

"Table 2, the unit of gestational age should be weeks."

**Authors' Response**: Thank you for capturing this. The unit has been corrected.

Line 253, should "increase" be "decrease"?

**Authors' Response**: The correct word is "increase." This accurately reflects that the gap between the intervention and control groups in terms of fear of childbirth scores widened as time progressed, indicating a stronger and more significant effect of the intervention. We appreciate your keen observation, and to avoid confusion, we have added a summary to emphasize this aspect. We also added a figure to visualize the change in FOC in the intervention and control groups in different time points of the study.

"Line 265, Should "Two" be "The"?"

**Authors' Response**: Thank you again for your input, and we have corrected the title.

Reviewer #3:

"Thank you for an interesting paper to review. I think it is a well-written paper with a novel and important approach for pregnant families. Nevertheless, the analysis seems to be relatively simple. I highly recommend using a logistic regression model as a minimum and then presenting your new results."

**Authors' Response**: Thank you sincerely for taking the time to review our manuscript.

We acknowledge your concern regarding our analysis. For our secondary outcome, we agree that we can add more analysis and have included a logistic regression to estimate the odds of CS in the intervention group compared to the control group. The results have been added to the manuscript. Regarding the primary outcome, FOC, we opted for a generalized linear model (repeated-measures ANOVA) due to the longitudinal nature of our data. This approach allowed us to analyze the outcome (FOC) measured over time on the same individuals at different time points (baseline, after 8 weeks, and after childbirth). The repeated-measures ANOVA effectively accounts for the correlation between these data points.

Furthermore, it is worth noting that ANOVA can indeed be seen as a specific case of regression analysis. In our study, we chose the repeated-measures ANOVA method because it offered us the ability to handle within-subject variability and gain valuable insights into the changes over time. By utilizing repeated-measures ANOVA, we were able to capture the temporal evolution of FOC in both the intervention and control groups.

While adding a regression analysis might yield similar results, we believe that the repeated-measures ANOVA was the most appropriate choice for our study's objectives and data structure. It allowed us to examine the effects of time and group on FOC, while also accounting for the repeated measurements within the same individuals.

We hope this explanation clarifies our analysis rationale, and we are open to any further suggestions or inquiries you may have.

**References**

1.	The statistics of patients at Labor and the increase in natural childbirth at Baharlou Hospital. 2009 [Available from: <http://194.225.51.22/baharlou>].

2.	Gharacheh M, Kalan ME, Khalili N, Ranjbar F. An increase in cesarean section rate during the first wave of COVID-19 pandemic in Iran. BMC Public Health. 2023;23(1):936.

3.	Shahshahan Z, Heshmati B, Akbari M, Sabet F. Caesarean section in Iran. The Lancet. 2016;388(10039):29-30.
