# Supplementary material for: The effect of an mHealth application based on continuous support and education on fear of childbirth, self-efficacy, and birth mode in primiparous women: A randomized controlled trial
Source: PLoS One. 2023 Nov 1;18(11):e0293815. doi: 10.1371/journal.pone.0293815 (PMC10619799; doi:10.1371/journal.pone.0293815)
Supplement: Attachment — Submitted filename: Response to Reviewers.docx [file pone.0293815.s005.docx]

**Reviewer #1:**

“Reviewer #1: The authors have done a good job in responding to the majority of my prior comments and I appreciate their efforts. There remain however, a couple of outstanding questions and suggestions.”
“1. Methods. It is important that the methods are described in such a way that the intervention could be replicated. After learning that this is a Master’s project, I am left wondering who and how the educational resources were created? - this is a very labor intensive effort. Are the videos, messages, and images all original content? I would like to know how many videos, images, texts were created or included? This can be quantified and added to table 1. If the program linked to any pre-existing videos or materials, they should be referenced.”

**Authors' Response:** Thank you for your comment. Over the past years, Iran has witnessed a range of initiatives aimed at producing health-related materials across various fields. The first author of this paper (SK) has actively participated in such endeavors, particularly in the field of midwifery, with the objective of providing her patients with the most up-to-date, evidence-informed recommendations. Additionally, although creating content for a mHealth application can be labor-intensive, these materials can be used many times, making mHealth applications more cost-effective in the long term. In this project, the research team meticulously curated and organized existing materials while also incorporating fresh information from diverse scientific sources, spanning from textbooks to comprehensive Cochrane systematic reviews. We have included the list of references used in the method section. It's important to note that we refrained from utilizing any pre-existing materials to uphold copyright integrity. The number of videos, images, and text messages has been added to Table 1.

“2. In the Methods section titled, “Continuous support and engagement of women:”, where the authors state (pg 11 line 205) “to provide women with an accessible source of information about their pregnancy and childbirth decisions"…The authors could clarify that the support did not include any prescription of medications or referrals for labs or testing of any kind.”

**Authors' Response:** Thanks for keen your comment. We have provided this important information to our “Continuous support and engagement of women” section.

Now it reads:

“However, this feature was not intended to substitute the usual antenatal care, and no medication prescriptions, requesting laboratory tests, or imaging were offered via our mHealth.”

“

“3. I understand that the project did not have the resources to analyze frequency and type of contacts (dose and content of support messaging) with healthcare providers. However, I think this is an important issue for discussion in the limitations/future research section to add a statement acknowledging that frequency and content of support from obstetric providers and use of messaging were not measured. If the authors are able to add any assessment in the limitations section about how frequently they were contacted- i.e. -on average providers estimated they were contacted 1-5 times per day, 20 times a day, once a week…..anything would help.”

**Authors' Response:** We have indeed recognized the importance of measuring the frequency and content of support received by participants during the intervention. We have added a paragraph in the limitations section to acknowledge the significance of this aspect. Since we did not collect precise data on the frequency of interactions with healthcare providers via the application, we refrained from providing specific statistics regarding the frequency of contact but instead emphasized its importance for future research. This key point has been highlighted in our discussion. We appreciate your insightful feedback, which has contributed to a more comprehensive understanding of the study's limitations and future research directions.

This section now reads:

“In this study we did not capture the frequency and nature of interactions between participants and healthcare providers who delivered the continues support feature of mHealth intervention. Consequently, the specific types of support, the average frequency of provider contact (such as interactions per day or week), and the potential impact of these interactions on maternal health outcomes remain unexplored in this study. Future research is needed to delve deeper into understanding the dynamics and potential benefits of such interactions for expecting mothers.”

4. On pg 20 line 349- Future research -need to consider not availability of “research personnel” but rather availability of qualified obstetric providers who can provide the education and advice needed.

**Authors' Response:** Thank you for capturing this. We now used the term 'sufficient qualified healthcare professionals in the field of obstetrics' to better convey our message.

**Reviewer #3**

“Reviewer #3: Thank you for addressing all of our comments. However, I believe that your statistical analysis could be more robust by utilizing logistic regression models instead of ANOVA. Your statistical advisor should be likely aware that a logistic regression can be employed for any longitudinal study to measure the effects while controlling for potential confounders. An alternative model you could use to adjust is a Poisson model. Although, you have made an effort to include one logistic regression model in your analysis. Just make sure to incorporate a table presenting these results. This will ensure clarity on the variables you've included for adjustment. If the limits on tables/word count prevent this, consider at least mentioning in your results section which variables you used for adjustment and add this table in the Supporting information section.”

**Authors' Response:** Thank you for your meticulous feedback on our analysis and results section. We have significantly revised the results section to incorporate your suggestions. Now we have presented the result of regression models for all of our outcomes.

To elaborate on our rationale for employing a linear regression model in our analyses, as opposed to logistic regression, we considered the continuous nature of the outcome variables (fear of childbirth and self-efficacy score). Given that our study's primary objective was to capture the degree of change in fear of childbirth and self-efficacy scores, we opted not to categorize our outcome or investigate the intervention's effect on change between categories. This approach allowed us to better account for the variability of outcomes within groups. Additionally, there is no established threshold to categorize self-efficacy from a continuous measure into a categorical variable [1]. Previous research has also treated fear of childbirth and self-efficacy as continuous variables, ensuring improved comparability of our results with existing literature.

Regarding adjustments in our model, the randomization in our study design was designed to balance known and unknown confounders between the two groups. Furthermore, our descriptive analysis in Table 1 revealed no statistically significant differences in known confounders such as age, education, and income levels, and the minor variations between groups were not a cause for concern. In addition, baseline scores for fear of childbirth and self-efficacy were balanced. This suggests the success of our randomization process. Therefore, initially, we presented unadjusted models in our results, as these confounders were anticipated to have a negligible impact on the outcomes [2]. However, in line with your suggestion, we conducted adjusted models accounting for age, income, education, and baseline outcome scores, and we have included this information in the supplementary material. Additionally, in the results section, we have clarified which variables were used for adjusted models, highlighting that the results are presented with these variables held constant.

1- Naggara, O., Raymond, J., Guilbert, F., Roy, D., Weill, A., & Altman, D. G. (2011). Analysis by categorizing or dichotomizing continuous variables is inadvisable: an example from the natural history of unruptured aneurysms. AJNR. American journal of neuroradiology, 32(3), 437–440. https://doi.org/10.3174/ajnr.A2425

2- Vickers A J, Altman D G. Analysing controlled trials with baseline and follow up measurements BMJ 2001; 323 :1123 doi:10.1136/bmj.323.7321.1123

“In line 227: Please consider to add the words "A logistic regression MODEL was also PERFORMED to estimate the odds of CS in the intervention group..." to complete your sentence.”

**Authors' Response:** Thank you for capturing this. We have revised it accordingly.
